# The potential of whole genome sequencing in pharmacogenetics: a retrospective health record study in rare disease patients

**DOI:** 10.1038/s41431-026-02025-w

**Published:** 2026-02-04

**Authors:** Madeline Gorny, Katja S. Just, Tim Krüger, Matthias Begemann, Florian Kraft, Thomas Eggermann, Jeremias Krause, Miriam Elbracht

**Affiliations:** 1 Center for Human Genetics and Genomic Medicine, Medical Faculty, RWTH Aachen University, Aachen, Germany; 2 Institute of Clinical Pharmacology, Medical Faculty RWTH Aachen University, Aachen, Germany

**Keywords:** Genetics research, Adverse effects

## Abstract

The clinical relevance of pharmacogenetics (PGx) is becoming increasingly evident as knowledge in this field expands. As of May 2025, 209 clinical guideline annotations are already listed on the internationally recognized ClinPGx website. Nevertheless, except for a few indications, the implementation of PGx in clinical practice currently remains limited in most countries. At the same time, whole genome sequencing (WGS) is increasingly applied in clinical diagnostics, particularly for rare and oncological diseases. These data could also be used for simultaneous PGx analysis. In a retrospective study, we analysed short-read WGS data from 1,000 individuals, including index patients with suspected rare disorders and their relatives. For a subset of 359 individuals, medical reports were reviewed to document drug prescriptions. Guidelines published by PGx consortia on ClinPGx were used for phenotype assignment and interpretation. Clinically relevant PGx variants were detected in 97% (*n* = 970) of the cohort. Among patients with drug prescriptions (*n* = 359), 30% (*n* = 111) had been prescribed at least one medication for which their PGx profile would recommend therapy adjustment. Additionally, CNVs and rare variants were detected, which in 28% (*n* = 8) resulted in modified therapeutic recommendations. While the most (cost)-efficient strategy for broad PGx implementation remains subject of future research, our findings demonstrate that existing WGS data, such as those generated in the context of rare disease patients, could provide substantial benefits for PGx diagnostics with minimal additional effort.

## Introduction

As a field of precision medicine (also known as individualised or personalised medicine), pharmacogenetics (PGx) has gained increasing importance in recent decades. PGx examines how a patient’s genetic profile influences their response to therapy and the occurrence of adverse drug reactions (ADRs)[1]. The broad potential of implementing pharmacogenetic testing has been documented previously, for targeted testing approaches[2], as well as for whole-genome-sequencing reaching back to as far as 2014[3–6].

Although the definition and calculation of costs of ADRs vary, several studies suggest that ADRs represent a significant economic burden[7–9]. The 2023 published PREPARE study by the Ubiquitous Pharmacogenomics (U-PGx) Consortium demonstrated a significant reduction of 30% of ADRs by preventive, panel-based pharmacogenetic testing and available treatment guidelines[2]. The systematic review by Morris et al. found that approximately three-quarters of the included studies reported pre-emptive pharmacogenetic testing to be cost-effective or cost-saving[10].

Although there is considerable interest in the use of PGx among pharmacists and physicians, barriers remain, including challenges such as the standardization of PGx tests and their reported results, as well as a lack of information and knowledge among healthcare professionals[11, 12]. In recent years, consortia such as the Clinical Pharmacogenetics Implementation Consortium (CPIC; cpicpgx.org) and the Dutch Pharmacogenetics Working Group (DPWG; knmp.nl) have contributed to the implementation of scientific knowledge on relevant gene-drug interactions and genotype-phenotype assignment by compiling evidence and translating it into clinical guidelines. The Pharmacogenomics Knowledge Base (PharmGKB) is a comprehensive, publicly accessible resource that collects, curates, and disseminates pharmacogenetic guidelines[13]. Since July 2025 PharmGKB moved in to ClinPGx (clinpgx.org), integrating the formerly PharmGKB, CPIC and PharmCAT projects. The potential clinical relevance of PGx is underscored by consensus in the literature that nearly every individual carries at least one pharmacogenetic variant which, according to available guidelines, would warrant a therapy adjustment (referred to as an “actionable variant”)[14–17]. Approximately one in four patients is prescribed a medication for which they carry a genotype that leads to an actionable gene-drug recommendation (referred to as an “actionable gene-drug pair”)[2, 18, 19]. Several providers offer pharmacogenetic panel tests, which primarily utilize either microarray-based genotyping or targeted sequencing using short-read NGS. While microarrays enable high sample throughput, their ability to detect complex structural variants, particularly those occurring in the *CYP2D6* gene, remains limited. In contrast, targeted NGS approaches can identify more complex structural variations. However, these methods share the same fundamental limitation as microarrays: they are restricted to predefined genomic regions and thus cannot provide the comprehensive whole-genome analysis offered by whole-genome sequencing (WGS). WGS offers the additional advantage of detecting both known and rare or novel pharmacogenetic relevant variants, thereby enabling integrated pharmacogenetic diagnostics[20, 21]. This is particularly relevant given that rare variants account for approximately 30–40% of the total observed variation in pharmacogenes[22].

Rare disease patients are a cohort of patients who are increasingly receiving WGS in routine diagnostics of the underlying disease. A disease in Europe is considered rare if it affects fewer than 1 in 2,000 individuals. Worldwide, 3.5–5.9% of the population, or approximately 263–446 million people, are affected by rare diseases[23]. As these individuals are often chronically ill, it is reasonable to assume that they are exposed to a particularly wide range of medications. Therefore, it can be hypothesized that this patient group would especially benefit from utilizing the already available WGS data for PGx diagnostics.

In this retrospective electronic health record study, we aimed to assess the potential benefit of pharmacogenetic testing, particularly the advantages of WGS in the context of rare disease patients.

## Materials and Methods

### Cohort

**Fig. 1:**
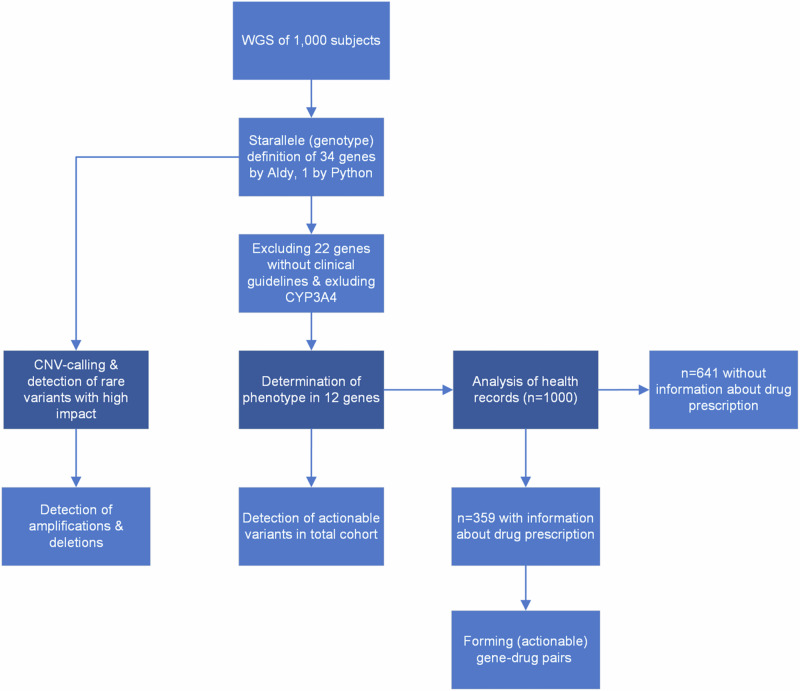
Study process. Methodological approach of the retrospective data analysis.

In this retrospective study we included a total of 1,000 individuals, consisting of index patients and their relatives, who underwent WGS as part of a genetic investigation for suspected rare genetic diseases. Fig. 1 shows the methodological approach in this study. Since both cohorts contain information about related individuals, we repeated the analysis for both cohorts with two additional cohorts, where only unrelated individuals were included. In general, we observed similar results, and the results of this analysis can therefore be found in the supplementary table S4, if not explicitly mentioned in the text.

### Genome sequencing and data processing

Library preparation was performed with Illumina DNA PCR-Free Prep according to the manufacturers protocol and used for direct sequencing. Short-read WGS was performed using the NovaSeq 6000 System (S4 Reagent Kit v1.5, Illumina Inc., San Diego, CA, USA) over 156 cycles. Alignment and variant calling were performed using the Illumina DRAGEN pipeline (v.07.021.645.4.0.3) using the hg38 reference genome. For pharmacogenomic annotation, genotyping of star alleles was conducted using Aldy (v.4.4). Aldy leverages a combinatorial approach to resolve complex pharmacogenetic haplotypes and accurately identifies star alleles, even in highly polymorphic regions. The tool integrates sequencing data with reference to pharmacogenetic databases to provide detailed allele calls, including Copy number variations (CNVs) and structural variants[24]. Annotating of the following 34 genes was performed by using Aldy, as these genes were supported by the version used in this study: *CFTR, COMT, CYP1A1, CYP1A2, CYP2A13, CYP2A6, CYP2B6, CYP2C19, CYP2C8, CYP2C9, CYP2D6, CYP2E1, CYP2F1, CYP2J1, CYP2R1, CYP2S1, CYP2W1, CYP3A4, CYP4A43, CYP3A5, CYP3A7, CYP4F2, DPYD, G6PD, GSTM1, GSTP1, IFNL3, NAT1, NAT2, NUDT15, TPMT, SCLO1B1, UGT2B7, VKORC1*. The output generated by Aldy was processed and formatted using the python library pandas[25]. To determine the pharmacogenetically relevant variant rs2231142 in *ABCG2*, which is not supported by Aldy version 4.4, we applied the same workflow used for the detection of rare variants as explained below. To conduct an additional CNV analysis for the 34 pharmacogenes, CNV calling was performed using CNVkit[26]. The resulting CNVs were subsequently visualized and analysed with CNVizard[27]. To reduce the number of false positive calls we opted to only include CNVs larger than two exons, as well as CNVs for which we were able to visualise split reads in the Integrative Genomics Viewer (IGV)[28]. Additional CNV calling was performed because Aldy annotated CNVs only for *CYP2D6* and *CYP2A6* in the used version. To identify rare SNVs (single nucleotide variants) or Indels (polymorphisms involving insertions or deletions) not included in the classified star alleles of CPIC or DPWG, we used bcftools (v.1.13)[29] for filtering of the VCF files produced by the DRAGEN pipeline to the relevant regions and the Ensemble Variant Effect Predictor (VEP) for annotating (v. 111.0)[30]. Finally, we used the python library pandas[25] to format the output and to perform additional filtering. The filter criteria for rare variants were the following: 1. Annotated as a high-impact variant by VEP 2. A general population frequency less than 1% (gnomAD 4.1)[31]. In our SNV-analysis, we focused on high impact variants (alterations with a substantial functional consequence) such as frame-shift-variants and stop-codon-variants. Further Data analysis and visualization, including the generation of graphs, was performed using Microsoft Excel (version16.94) and Microsoft Visio 2019.

### Variant analysis

In the further analysis, we excluded 22 of the 35 initially considered genes for which either no drug prescription guidelines were published by CPIC or DPWG on ClinPGx, or for which the guidelines stated “no recommendation” regarding therapy adjustment. Additionally, we did not include *CYP3A4*, because no CPIC-guideline is available for *CYP3A4* and it is mainly influenced by environmental factors. The remaining 12 genes *CYP2B6, CYP2C19, CYP2C9, CYP2D6, CYP3A5, DPYD, G6PD, NUDT15, TPMT, SCLO1B1, VKORC1* and *ABCG2* were included in our analysis. To detect actionable genetic variants in our cohort, defined as variants with available guidelines that result in therapy adjustment, a change in medication, or the recommendation for patient monitoring, we further determined the participants phenotype (i.e. type of metabolizer such as “normal metabolizer”). For phenotyping, we primarily used the comprehensive nomenclature provided by CPIC. In the following, we referenced the “guideline annotation table” on ClinPGx and examined whether there was at least one guideline for at least one drug that recommended a therapy adjustment for the respective phenotype. We focused exclusively on guidelines published by CPIC and DPWG. The subsequent analysis was performed in the subcohort. The subcohort comprised all participants for whom medication information was available. We reviewed all available participants’ health records to assess medication history. Specifically, we assumed that an individual had taken a drug at least once in their lives if it was documented in a clinical report from previous hospital stays, listed as an ongoing therapy or recommended as a future treatment. Gene-drug pairs (GDPs) were generated by combining each participants prescribed drug with their specific genotype-phenotype combination for the gene affecting efficacy and safety (e.g. involved in metabolism, coding transporter) of that drug. This was performed for all prescribed drugs in our cohort that matched one of the 79 medications listed in the “guideline annotation table” and were associated with one of the 12 aforementioned genes included in this analysis. A single drug may be represented in multiple GDPs, as several genes can be implicated in its metabolic pathways. To identify actionable GDPs, defining a GDP with an existing guideline for therapy adjustment recommendation, we used the drug specific drop-down tool under “Clinical Guideline Annotations” on ClinPGx. We distinguished between the following categories: “no action” (no therapy adjustment recommended), “dosing” (recommendation to adjust the dosage), “alternate drug” (recommendation for an alternative medication), “other” (a type of recommendation other than dosage adjustment or alternative medication) or a combination of two recommendations (e.g., “dosing/ alternate drug”). The categories “dosing”, “alternate drug” and “dosing/alternate drug” were considered as actionable, while recommendations classified as “other” were considered as actionable only based on their specific content. Furthermore, we investigated whether the phenotype and consequently the therapeutic recommendation for the individuals changed as a result of additional CNV or rare variant analysis, focussing on rare high-impact variants and copy number variants.

## Results

The study population consisted of a total of 389 families, each including one index patient and, in most cases, their family members. Overall, 1000 individuals underwent WGS. Drug prescription information was available for 359 (35.9%) of these individuals, forming our subcohort. The subcohort showed a comparable mean age and a similar sex-distribution compared to the whole group. The characteristics of the study population are shown in table S1 in the supplementary information.

### Phenotype frequencies

**Fig. 2:**
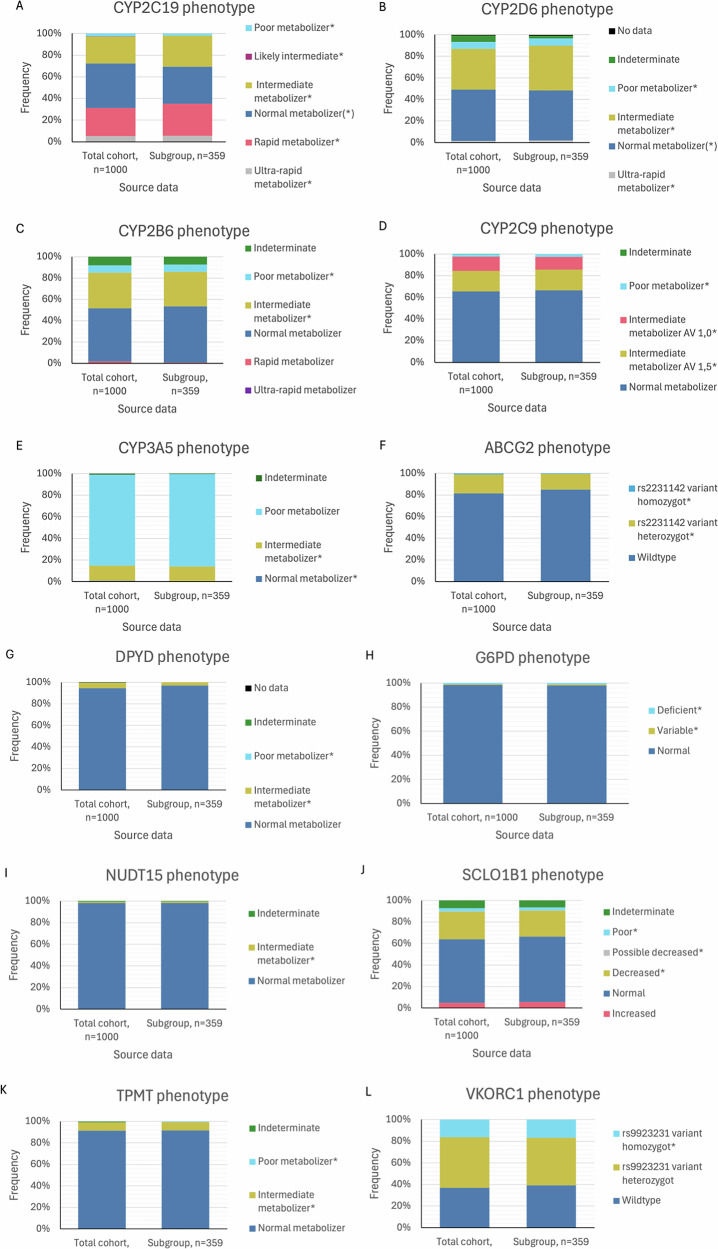
Relevant genes for applied pharmacogenetics. **A**–**L** shows the distribution of phenotypes and frequencies by genes in the total cohort of all participants and the subcohort with information of drug prescription. Phenotypes marked with an asterisk (*) are considered actionable.

Among all 12 genes analysed, 970 (97%) participants in the overall study population carried at least one actionable variant, based on current guidelines. Among the 389 unrelated index participants, 384 (98,7%) carried at least one actionable variant. The gene with the highest prevalence of actionable phenotypes was *CYP2C19*, affecting 589 individuals (58.9%) in the entire WGS cohort and 236 (65.7%) in the subcohort. This was followed by *CYP2D6* (entire cohort: *n* = 458, 45.8%; subcohort: *n* = 180, 50.1%) and *CYP2B6* (entire cohort: *n* = 401, 40.1%; subcohort: *n* = 141, 39.3%). Notably, if guidelines for pantoprazole (*CYP2C19*) and atomoxetine (*CYP2D6*) were applied, even normal metabolizer (NM) would receive a treatment recommendation. Consequently, all participants with available CYP2C19 data would be classified as having an actionable variant (*n* = 1000, 100% of the entire cohort; *n* = 359, 100% of the subcohort). Similarly, those with a clear CYP2D6 metabolizer status would also be classified as having an actionable variant (*n* = 935, 93.5% of the entire cohort; *n* = 347, 96.7% of the subcohort). In contrast, the lowest frequencies of clinically relevant phenotypes were observed in *NUDT15* (entire cohort: *n* = 11, 1.1%; subcohort: *n* = 4, 1.1%) and G6PD (entire cohort: *n* = 14, 1.4%; subcohort: *n* = 7, 1.9%). A detailed distribution of phenotypes and their frequencies by genes is provided in table S2 in the supplementary material. Fig. 2 shows the distribution of phenotypes and frequencies by genes in the total cohort and the subcohort.

### Subcohort (*n* = 359)

In the subcohort, 293 different medications were taken at least once by a patient. Ibuprofen was the most documented drug (n = 79, 22.0%), followed by pantoprazole (*n* = 77, 21.5%) and acetaminophen (paracetamol) (n = 63, 17.6%). On average, each patient took 5.4 ( ± 5.1) medications at least once. Of the 293 drugs reported in our cohort, 79 were also represented in the “guideline annotation table”, which includes a total of 206 drugs (as of May 2025). On average, individuals were prescribed 2.5 ( ± 2.0) of these guideline-relevant drugs.

Among the 79 medications included in the “guideline annotation table”, 49 were associated with specific star-allele-based prescribing recommendations, rather than being designated with ‘no recommendation’ status. Individuals were prescribed on average 2.0 ( ± 1.3) of these drugs.

### Gene-drug pairs

**Fig. 3:**
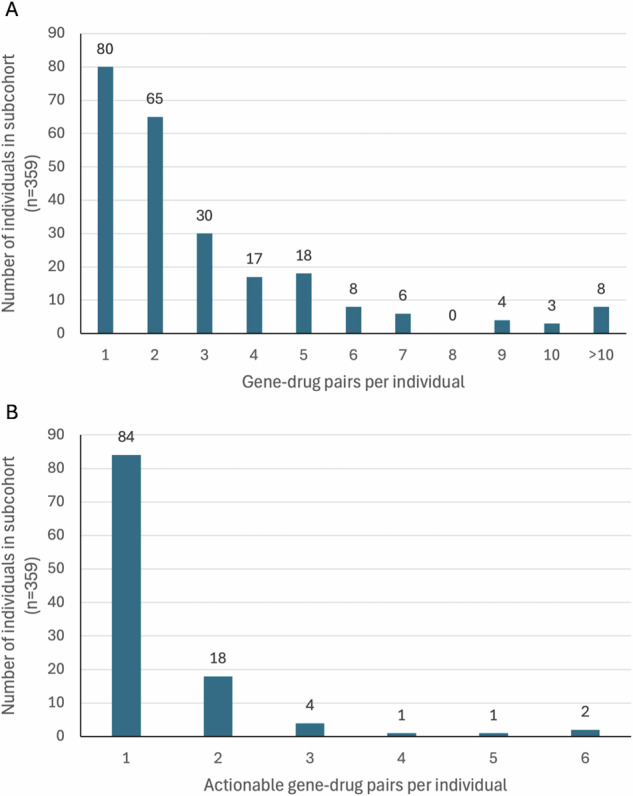
Gene-drug pairs (GDPs) per individual with information of prescribed medications (subcohort: *n* = 359). **A** illustrates the number of GDPs per individual and **B** actionable-GDPs per individual.

We identified 713 GDPs across 233 individuals. On average, each individual harboured 3.0 (±2.6) GDPs. Fig. 3 illustrates the distribution of GDPs per Individual.

### Actionable gene-drug pairs

In total, 110 individuals (30.6%) carried at least one actionable GDP. On average one individual harboured 1.4 (±0.9) actionable GDPs. In aggregate, 153 GDPs were actionable and would therefore lead to therapy adjustments. Figure 3B shows the distribution of actionable GDPs per individual.

*CYP2C19* exhibited the highest number of clinically relevant interactions: Among 137 GDPs with available guideline-based recommendations, 105 (76.6%) were actionable. The most actionable GDPs involved pantoprazole (*n* = 77) and omeprazole (*n* = 10). CYP2D6 ranked next, with 24 (22.6%) out of 106 GDPs deemed actionable, most commonly involving amitriptyline (*n* = 5). The lowest incidence of actionable GDPs, with zero cases, was observed for *ABCG2*, *DPYD, G6PD, NUDT15* and *TPMT*. A full breakdown of actionable gene-drug pairs per gene is provided in Table 1. We also observed that combining the genotypes of two genes involved in the safety and efficacy of a prescribed drug led to different recommendations compared to the consideration of a single gene alone. A breakdown for these gene-gene drug pairs is provided in table S3 in the supplementary information.

**Table 1:** Incidence of actionable GDPs in the subcohort (*n* = 359).

Drug	Frequency drug-gene pair with recommendation, (*n* = 406)	Phenotype	Frequen cy per phenoty pe	Recommendation (ClinPGx based on CPIC/DPWG guidelines)	Frequencies of recommendation with action, *n* (%)
***CYP2C19***	**137 (33.7)**				**105 (76.6)**
Amitriptyline	10 (7.3)				3 (30)
		UM	-	Alternate drug/ dosing	
		RM	3	Alternate drug/ dosing	
		NM	1	No action	
		IM	6	No action	
		PM	-	Alternate drug/ dosing	
(Es)citalopra m	10 (7.3)				7 (70)
		UM	-	Alternate drug/ dosing	
		RM	4	Alternate drug/ dosing	
		NM	3	No action	
		IM	3	Dosing	
		PM	-	Alternate drug/ dosing	
Clopidogrel	3 (2.2)				1 (33.3)
		UM	-	No action	
		RM	1	No action	
		NM	1	No action	
		IM	1	No action	
		PM	-	Alternate drug	
Doxepin	2 (1.5)				1 (50)
		UM	1	Alternate drug/ dosing	
		RM	-	Alternate drug/ dosing	
		NM	-	No action	
		IM	1	No action	
		PM	-	Alternate drug/ dosing	
Omeprazole	16 (11.7)				10 (62.5)
		UM	-	Dosing	
		RM	4	Dosing	
		NM	6	No action	
		IM	6	Dosing	
		PM	-	Dosing	
Pantoprazole	77 (56.2)				77 (100)
		UM	6	Dosing	
		RM	22	Dosing	
		NM	24	Dosing	
		IM	21	Dosing	
		PM	4	Dosing	
Sertraline	13 (9.5)				4 (30.8)
		UM	-	No action	
		RM	2	No action	
		NM	7	No action	
		IM	4	Dosing	
		PM	-	Alternate Drug/Dosing	
Trimipramine	4 (2.9)				2 (50)
		UM	-	No action	
		RM	2	Alternate Drug/Dosing	
		NM	-	No action	
		IM	2	No action	
		PM	-	Alternate Drug/Dosing	
Voriconazole	2 (1.5)				0 (0)
		UM	-	Alternate drug	
		RM	-	Alternate drug	
		NM	1	No action	
		IM	1	No action	
		PM	-	Alternate drug	
***CYP2D6***	**106 (26.1)**				**24 (22.6)**
Amitriptyline	10 (9.4)				5 (50)
		UM	1	Alternate drug	
		NM	4	No action	
		IM	4	Dosing	
		PM	-	Alternate drug	
		indeterminate	1		
Aripiprazole*	6 (5.7)				2 (33.3)
		UM	-	Alternate drug	
		NM	3	No action	
		IM	1	Dosing	
		PM	1	Alternate drug	
		indeterminate	1		
Atomoxetine	3 (2.8)				3 (100)
		UM	-	Dosing	
		NM	3	Dosing	
		IM	-	Dosing	
		PM	-	Dosing	
Doxepin	2 (1.9)				1 (50)
		UM	-	Alternate drug	
		NM	1	No action	
		IM	1	Dosing	
		PM	-	Alternate drug	
Flecainide*	4 (3.8)				3 (75)
		UM	-	Alternate drug	
		NM	1	No action	
		IM	3	Dosing	
		PM	-	Alternate drug	
Haloperidol*	3 (2.8)				0 (0)
		UM	-	Alternate drug/dosing	
		NM	2	No action	
		IM	1	No action	
		PM	-	Dosing	
Metoprolol	20 (18.9)				0 (0)
		UM	1	No action	
		NM	11	No action	
		IM	7	No action	
		PM	-	Alternate drug	
		indeterminate	1		
Ondansetron	13 (12.3)				1 (7.7)
		UM	1	Alternate drug	
		NM	6	No action	
		IM	4	No action	
		PM	2	No action	
Propafenone*	1 (0.9)				0 (0)
		UM	-	Alternate drug/dosing	
		NM	1	No action	
		IM	-	Alternate drug/dosing	
		PM	-	Dosing	
Risperidone*	24 (22.6)				1 (4.2)
		UM	-	Alternate drug/dosing	
		NM	11	No action	
		IM	10	No action	
		PM	1	Dosing	
		Indeterminate	2		
Tamoxifen	2 (1.9)				2 (100)
		UM	-	No action	
		NM	-	No action	
		IM	2	Alternate drug/dosing	
		PM	-	Alternate drug/dosing	
Tramadol	8 (7.6)				2 (25.0)
		UM	-	Alternate drug	
		NM	6	No action	
		IM	2	Other**	
		PM	-	Alternate drug	
Trimipramine	4 (3.8)				3 (75)
		UM	-	Alternate drug/dosing	
		NM	1	No action	
		IM	3	Dosing	
		PM	-	Alternate drug/dosing	
Venlafaxine	5 (4.7)				0 (0)
		UM	-	No action	
		NM	2	No action	
		IM	3	No action	
		PM	-	Alternate drug	
Zuclopenthixol*	1 (0.9)				1 (0)
		UM	-	Dosing	
		NM	-	No action	
		IM	-	Dosing	
		PM	1	Dosing	
***CYP2C9***	**86 (21.2)**				**12 (14.0)**
Celecoxib	6 (7.0)				1 (16.7)
		NM	2	No action	
		IM (AV*1.5)	3	No action	
		IM (AV* 1)	1	Dosing	
		PM		Dosing	
Ibuprofen	79 (91.9)				11 (14.0)
		NM	53	No action	
		IM (AV*1,5)	15	No action	
		IM (AV* 1)	10	Dosing	
		PM	1	Alternate drug/Dosing	
Phenytoin	1 (1.1)				0 (0)
		NM	1	No action	
		IM (AV*1.5)	-	No action	
		IM (AV* 1)	-	Dosing	
		PM	-	Dosing	
***CYP2B6***	**13 (3.2)**				**6 (46.2)**
Sertraline	13 (100)				6 (46.2)
		UM	-	No action	
		RM	-	No action	
		NM	6	No action	
		IM	4	Dosing	
		PM	2	Alternate Drug/dosing	
		indeterminate	1	No action	
***SCLO1B1***	**26 (6.4)**				**6 (23.1)**
Atorvastatin	13 (50)				2 (15.4)
		Increased function	1	No action	
		Normal function	10	No action	
		Decreased function	1	Dosing	
		Poor function	1	Alternate drug/dosing	
Lovastatin	1 (3.8)				0 (0)
		Increased function	-	No action	
		Normal function	1	No action	
		Decreased function	-	Alternate drug/Dosing	
		Poor function	-	Alternate drug	
Pravastatin	1 (3.8)				0 (0)
		Increased function	-	No action	
		Normal function	1	No action	
		Decreased function	-	Other**	
		Poor function	-	Alternate drug/dosing	
Rosuvastatin	3 (11.5)				2 (66)
		Increased function	-	No action	
		Normal function	1	No action	
		Decreased function	-	Other**	
		Poor function	2	Dosing	
Simvastatin	8 (30.8)				2 (25)
		Increased function	1	No action	
		Normal function	5	No action	
		Decreased function	2	Alternate drug/dosing	
		Poor function	-	Alternate drug	
***ABCG2***	**7 (1.7)**				**0 (0)**
Allopurinole*	4 (57.1)				0 (0)
		Wildtype	4	No action	
		rs2231142 variant homozygote	-	Dosing	
		rs2231142 variant heterozygote	-	Dosing	
Rosuvastation	3 (42.9)				0 (0)
		Wildtype	2	No action	
		rs2231142 variant homozygote	-	Alternate drug/dosing	
		rs2231142 variant heterozygote	1	No action	
***CYP3A5***	**4 (1.0)**				**0 (0)**
Tacrolimus	4 (100)				0 (0)
		NM	-	Dosing	
		IM	-	Dosing	
		PM	4	No action	
***DPYD***	**1 (0.2)**				**0 (0)**
(5-) Fluorouracil	1 (100)				0 (0)
		NM	1	No action	
		IM	-	Dosing	
		PM	-	Alternate drug	
***G6PD***	**3 (0,7)**				**0 (0)**
Dapsone	2 (66.7)				0 (0)
		Normal	2	No action	
		Variable	-	Other**	
		Deficient	-	Alternate drug	
Nitrofurantoin	1 (33.3)				0 (0)
		Normal	1	No action	
		Variable	-	Other**	
		Deficient	-	Other**	
***NUDT15***	**11 (2.7)**				**0 (0)**
Azathioprine	3 (27.3)				0 (0)
		NM	3	No action	
		IM	-	Dosing	
		PM	-	Alternate Drug/dosing	
Mercaptopuri ne	5 (45.5)				0 (0)
		NM	5	No action	
		IM	-	Dosing	
		PM	-	Alternate Drug/dosing	
Thioguanine	3 (27.3)				0 (0)
		NM	3	No action	
		IM	-	Dosing	
		PM	-	Alternate Drug/dosing	
***TPMT***	**11 (2.7)**				**0 (0)**
Azathioprine	3 (27.3)				0 (0)
		NM	3	No action	
		IM	-	Dosing	
		PM	-	Alternate Drug/dosing	
Mercaptopuri ne	5 (45.5)				0 (0)
		NM	5	No action	
		IM	-	Dosing	
		PM	-	Alternate Drug/dosing	
Thioguanine	3 (27.3)				0 (0)
		NM	3	No action	
		IM	-	Dosing	
		PM	-	Alternate Drug/dosing	
***VKORC1***	**1 (0.2)**				**0 (0)**
Phenprocoumon	1 (100)				0 (0)
		Wildtyp	1	No action	
		rs9923231 variant heterozygo us	-	No action	
		rs9923231 variant homoozygote	-	Dosing	

### CNV-Calling

#### Deletions

**Fig. 4:**
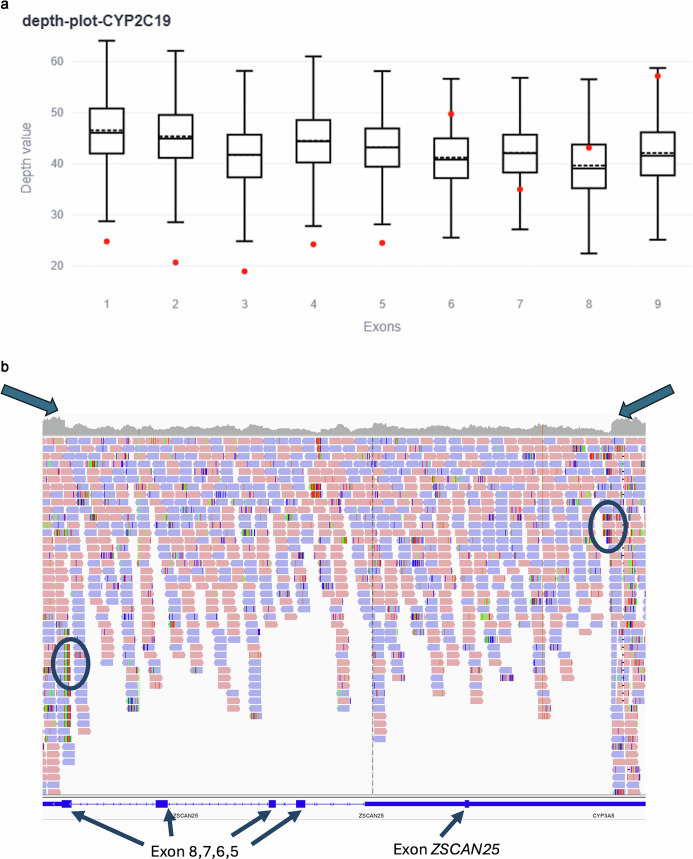
Copy number variation (CNV) detection using the tools CNVizard and IGV-Browser. **a** illustrates the deletion of first 5 exons in *CYP2C19* by CNVizard. In the boxplots generated by CNVizard, outliers in read depth (depicted as red dots) are observable within the first five boxplots, corresponding to the deletion of the affected exons. **b** illustrates a gene section of CYP3A5 with the deletion of exons 5, 6 and 7 with breaking points in exon 8 by the IGV Browser. A pronounced reduction in read depth coverage is evident in the IGV-browser, which is indicative of a (heterozygous) deletion (marked with blue arrows above). In addition, the breakpoints of the deletion can be identified by colourful split reads (marked with blue circles). In the lower panel, indicated by blue arrows, the exons are marked with blue boxes. An overlap with the ZSCAN25 is visible (highlighted by the right most blue arrow).

Within the entire cohort, 12 individuals, 9 of whom were unrelated; were found to harbour deletions in pharmacogenes other than *CYP2D6* all of which are likely to be clinically relevant. In six (50%) of these individuals, of whom 5 were unrelated, detection of the deletion resulted in a change in phenotype and therapy recommendation. Seven individuals carried a deletion affecting the first five exons of *CYP2C19*. Five of these were unrelated individuals (allele frequency (AF) in total cohort: 0.7%; allele counts in cohort (AC): 7/1000; AF in unrelated cohort: 0.77%; AC in unrelated cohort: 5/650). In Fig. 4 we demonstrate the process of identifying CNVs using CNVizard and the IGV-Browser.

Of these seven individuals, two were identified as ultra-rapid metabolizers (UMs) (*17/*17) and four as normal metabolizers (NMs) (*1/*1) by Aldy, while it can be assumed that they all resulted in an IM phenotype due to the deletion of one allele and therefore the therapy recommendation for at least one drug would change. The seventh individual was identified as poor metabolizer (PM) by Aldy. A deletion in one allele would not have changed the phenotype or the therapy recommendation. In *COMT* we additionally observed three whole gene deletions, all in unrelated individuals diagnosed with the microdeletion syndrome (22q11.2), indicating that the *COMT* deletion was part of a larger structural variant (AF in total cohort: 0.3%; AC in total cohort: 3/1000; AF in unrelated cohort: 0.46%; AC in unrelated cohort: 3/650). Currently, there is insufficient evidence to establish a relationship between drug efficacy and safety in relation to *COMT*. Followingly, no official guideline is available. Finally, we observed a CNV affecting the *CYP3A5* gene in two related individuals (AF in total cohort: 0.2%; AC in total cohort: 2/1000; AF in unrelated cohort: 0.15%; AC in unrelated cohort: 1/650). In these two individuals, we observed a deletion encompassing the complete exons 5,6,7, in which the breaking point was found in exon 8 (transcript CYP3A5 NM_000777.5), likely resulting in an impaired function of the corresponding protein. Since both individuals had previously been classified as PM, there would be no change in the therapy recommendation. In our cohort, 5 of the 12 individuals with a deletion were part of the subcohort with available drug prescription data. Two individuals were prescribed drugs for which the gene containing the deletion was relevant for the drugs´ safety and efficacy. One of these individuals had a deletion in *COMT* and was prescribed methylphenidate. The Aldy output suggested that the remaining allele was a Val-allele. Since there is insufficient evidence regarding the gene-drug interaction between *COMT* and methylphenidate, no conclusion can be drawn about the effect of the deletion in this case [32]. The second individual harboured a deletion in *CYP2C19* and was prescribed pantoprazole. For this individual, Aldy detected the star allele *2B homozygote, which already classified the proband as a PM. Therefore, the additional CNV calling would not have led to any changes in medication.

#### Amplifications

In the total cohort, we detected five whole-gene amplifications in pharmacogenes other than *CYP2D6*. Four of these participants were unrelated. In two of these individuals (40%), additional CNV calling led to a modification of the phenotype; in one individual (20%), this also resulted in a change in therapy recommendation. Three individuals showed a duplication in *COMT*; of these, two related individuals were heterozygous (*Met/*Val) (AF in total cohort: 0.2%; AC in total cohort: 2/1000; AF in unrelated cohort: 0.15%; AC in unrelated cohort: 1/650) and one was homozygous for *Met (associated with low activity) (AF in total cohort: 0.1%; AC in total cohort: 1/1000; AF in unrelated cohort: 0.15%; AC in unrelated cohort: 1/650). As previously mentioned, there is currently no guideline for *COMT*. A duplication in *CYP2B6* was found in one individual, initially classified as an IM with the genotype *1/*6 (AF in total cohort: 0.1%; AC in total cohort: 1/1000; AF in unrelated cohort: 0.15%; AC in unrelated cohort: 1/650). The *6 allele is associated with decreased function. If this allele were affected by the duplication, the phenotype would likely have resulted in a NM and therefore in a different therapy recommendation. Duplication of the normal-function allele *1 in combination with the *6 allele might also have led to a normal or slightly increased metabolic rate. In *CYP2C9*, a whole-gene duplication was found in one individual (AF in total cohort: 0.1%; AC in total cohort: 1/1000; AF in unrelated cohort: 0.15%; AC in unrelated cohort: 1/650), primarily identified as a NM (*1/*1). Therefore, the phenotype likely shifted to a rapid metabolizer (RM). According to ClinPGx, no star allele combination is currently described that results in a RM or UM phenotype.

#### Rare variants

In 12 individuals, of whom 7 were unrelated, we identified at least one additional rare variant rated as high-impact variant by VEP, indicating a severe functional effect and potential pathogenicity. For one individual (8.3%), the additional information regarding the rare variant led to a modification of both the phenotype and the therapy recommendation. In two unrelated individuals (16.7%), it could potentially have influenced the phenotype and the therapy recommendation. In *CYP3A5* eight individals carried a frameshift mutation, two related individuals with (rs753330469; NM_0007.5:c.247dup (hg38)) (AF in total cohort: 0.2%; AC in total cohort: 2/1000; AF in unrelated cohort: 0.15%; AC in unrelated cohort: 1/650) and six with (rs54725341; NM_000777.5:c.1372del (hg38)) (AF in total cohort: 0.6%; AC in total cohort: 6/1000; AF in unrelated cohort: 0.46%; AC in unrelated cohort: 3/650). All were initially identified as a PM with the genotype *3/*3 by Aldy, which is the most common genotype in the European population and results in a nonfunctional protein[33]. The observed frameshift mutations would also likely have caused loss of function of the corresponding protein and therefore would not have altered the phenotype or therapy recommendation. Two related probands harboured the variant (rs201260783; NM_000777.5:c.746 T > A (hg38)), leading to a premature stop codon in *CYP3A5* (AF in total cohort: 0.2%; AC in total cohort: 2/1000; AF in unrelated cohort: 0.15%; AC in unrelated cohort: 1/650). One was identified as an IM with the genotype *1/*3. Depending on which allele was affected, the deletion would likely have resulted in either little change in phenotype or manifest as PM phenotype – which would change the therapeutic recommendation (e.g., for tacrolimus). In *CYP2C19*, one individual harboured a variant (rs374036992; NM_000769.4:c.463 G > T (hg38)) leading to a premature stop codon, while Aldy identified them as a RM with the genotype *1/*17 (AF in total cohort: 0.1%; AC in total cohort: 1/1000; AF in unrelated cohort: 0.15%; AC in unrelated cohort: 1/650). A stop codon in either allele would have resulted in an IM in both cases and change the therapy recommendation for at least one drug. Another individual exhibited a frameshift mutation in *SCLO1B1* (rs547253411; NM_006446.5:c.1925_1929 (hg38))(AF in total cohort: 0.1%; AC in total cohort: 1/1000; AF in unrelated cohort: 0.15%; AC in unrelated cohort: 1/650). For this index patient, Aldy could not provide a definitive result, indicating either *1/*20 (NM) or *19/*37 (*19 indeterminate function, *37 normal function). Taken into account the loss of function allele, the phenotype would have resulted in a decreased *SLCO1B1* function.

## Discussion

### Actionable pharmacogenetic variants affecting a large proportion of patients

In this retrospective electronic health record study, we were able to demonstrate the potential clinical relevance of using WGS data for individual treatment modifications. While we used a cross-sectional analysis, this may show the potential for further use of this data in the sense of a pre-emptive PGx diagnostics. In our cohort, actionable variants are considered clinically relevant for almost one-third of the subcohort, as these individuals received a drug for which there was a therapy recommendation (e.g., dose adjustment, drug change or caution during initiation and close monitoring). In consensus with our data, recent studies have identified a real-world prevalence of 23–31% for actionable GDPs among individuals receiving medication [2, 17, 34]. Our finding that 97% of the individuals exhibited an actionable variant aligns with the literature[2, 14, 19].

### Integrating rare variants and CNVs in pharmacogenomic testing

By leveraging short-read WGS data, we identified 29 individuals (2.9%) in the total cohort (20 individuals (3.1%) in the unrelated cohort) with either a CNV or a rare variant. Almost 30% (8/29) (35% (7/20) unrelated individuals) led to a change in phenotype and treatment recommendation. Our findings that CNVs in pharmacogenes other than *CYP2D6* are generally rare ( < 1%) align with the existing literature[35]. However, little is known about the frequency of CNVs in the pharmacogenes in which we detected variants. The unrelated allele frequency of the observed deletion of exons 1–5 in *CYP2C19* was with 0.77%, markedly higher than previously reported[35]. Known as *37, this CNV results in an unexpressed protein and was not included in Aldy and ClinPGx does not provide frequency data. Originally described by Botton et al. (2019) using microarray data from >78,800 cases, its frequency was estimated at 1:2000, classifying *37 as rare[35]. Our data suggest *CYP2C19* structural variants may be more frequent than assumed. In gnomAD, the total frequency is 0.14%, with the highest in the Finnish population (1.5%). Previous studies have shown CNV frequencies can differ from those found using NGS[36, 37]. The potential clinical relevance of CNVs such as *37 in *CYP2C19* is exemplified by six individuals whose predicted phenotype by Aldy shifted from UM or NM to IM detecting additional CNVs: While NM are advised to start with the standard dose of clopidogrel, patients with acute coronary syndrome or percutaneous coronary intervention who are intermediate metabolizers (IMs) are associated with an increased risk of adverse events under clopidogrel and should avoid this treatment[38]. Amplifications were also infrequent, with AFs < 1% in *COMT*, *CYP2C9* and *CYP2B6*. The *CYP2C9* duplication frequency in unrelated individuals (0.15%) diverged from Botton et al., who reported an allele frequency 30 times lower (0.005%)[35]. Compared to the literature, we found a lower frequency of rare, potentially deleterious variants with 0.11% (7/650) in unrelated cases[19, 39]. Gordon et al. found such variants in 12 *CYP* genes in about 9% of 6,503 individuals[39]. This difference reflects their broader inclusion of missense variants, whereas we considered only high impact variants. In our cohort, we observed variant frequencies partly consistent with those reported in gnomAD (e.g., *CYP2C19* exon1-5 deletion) but also identified unreported variants (e.g., *CYP3A5* exon 5-7 deletion with breaking point in exon 8). This was a deliberate design choice, as the evaluation of variants of uncertain clinical significance, that do not fall into the category of high-impact variants (e.g., missense variants), comes with several limitations. A possible limitation is that current in-silico prediction algorithms are less suitable to deal with less evolutionary conserved regions which is the case for multiple pharmacogenes[20]. Furthermore, current tools are often better in detecting loss-of functions variants compared to gain-of function variants, further complicating ranking of pharmacogenetically relevant genetic variants[20, 40]. Specialized algorithms for ranking pharmacogenetically relevant variants will be of high interest for whole-genome pharmacogenomic analysis, first algorithms for variants affecting the protein coding regions of pharmacogenes have been suggested[41]. As targeted diagnostics may only detect known variants and may fail to identify already known and potentially clinically relevant alterations[42], our findings underscore the added value of WGS for comprehensive genetic diagnostic. While clinically relevant SNVs with AF < 1% (e.g., *2 A in *DPYD*) are routinely tested, our data demonstrate that additional potentially clinically relevant variant of similar frequency, not considered by targeted diagnostic approaches, exist and can be detected with WGS.

### Pharmacogenomics in the rare disease context and beyond

Performing WGS once and enabling reuse of the resulting data as new insights emerge may facilitate potential cost savings. While WGS still carries a substantial price tag, the exponential cost reduction seen since its inception, combined with emerging AI-based analysis tools, anticipates broader accessibility. In contrast to panel-based PGx, the WGS offers the potential for a more comprehensive analysis of structural variants as well as newly identified variants in the future. In this study, we focussed on whole genome data available for rare diseases patients and their relatives, thereby using whole genome data which is already available from different diagnostic procedures and can followingly be used with minimal additional cost. Furthermore, rare disease patients are usually individuals that undergo a long diagnostic odyssey and get frequently admitted to the hospital or receive long time medications. Hence, we expect those patients potentially benefitting from the PGx analysis of available WGS data.

### Limitations

A limitation of our study is the absence of data on individuals’ ethnicity. Ethnic differences can influence the prevalence of potentially clinically relevant variants[43]. Furthermore, our total cohort does not consist of independent individuals but rather 389 index patients and their relatives. In the subcohort, the proportion of index cases was higher than in the overall cohort, which may introduce bias, as the index cases in our study were chronically ill and therefore potentially exposed to a greater number of medications. To mitigate these limitations, we repeated all analysis using two unrelated subcohorts which can be found in the supplementary material.

In summary, our study demonstrates that PGx testing using short-read WGS could provide substantial benefits. While CNVs and rare variants occur considerably less frequent than actionable variants, they can be clinically relevant. Consequently, the cost-effectiveness of pre-emptive PGx testing via WGS rather than panel-testing remains an important subject for further research. In an era where precision medicine is gaining increasing significance, a single WGS performed could represent a future vision for individualized medicine. Until such approaches become routine, existing WGS data, such as those generated for individuals with rare diseases, as in our study, could be utilized for pharmacogenetic diagnostics.

## Supplementary information

Supplementary_figure_S1

Supplementary_Figure_S2

Supplementary_figure_S2A

Supplementary_figure_S2B

Supplementary_figure_S3

Supplementary_figure_S3A

Supplementary_figure_S3B

Supplementary_Table_S1_PGx

Supplementary_Table_S2_PGx

Supplementary_Table_S3_PGx

Supplementary_tableS4_PGx

## Data Availability

The gene-drug pairs generated and used in this study are available in Table 1 and Supplementary table 2. Additional information is available from the corresponding author on reasonable request.
