# Supplementary figures and images for: The potential of whole genome sequencing in pharmacogenetics: a retrospective health record study in rare disease patients
Source: Eur J Hum Genet. 2026 Feb 4;34(5):691–703. doi: 10.1038/s41431-026-02025-w (PMC13171899; doi:10.1038/s41431-026-02025-w)

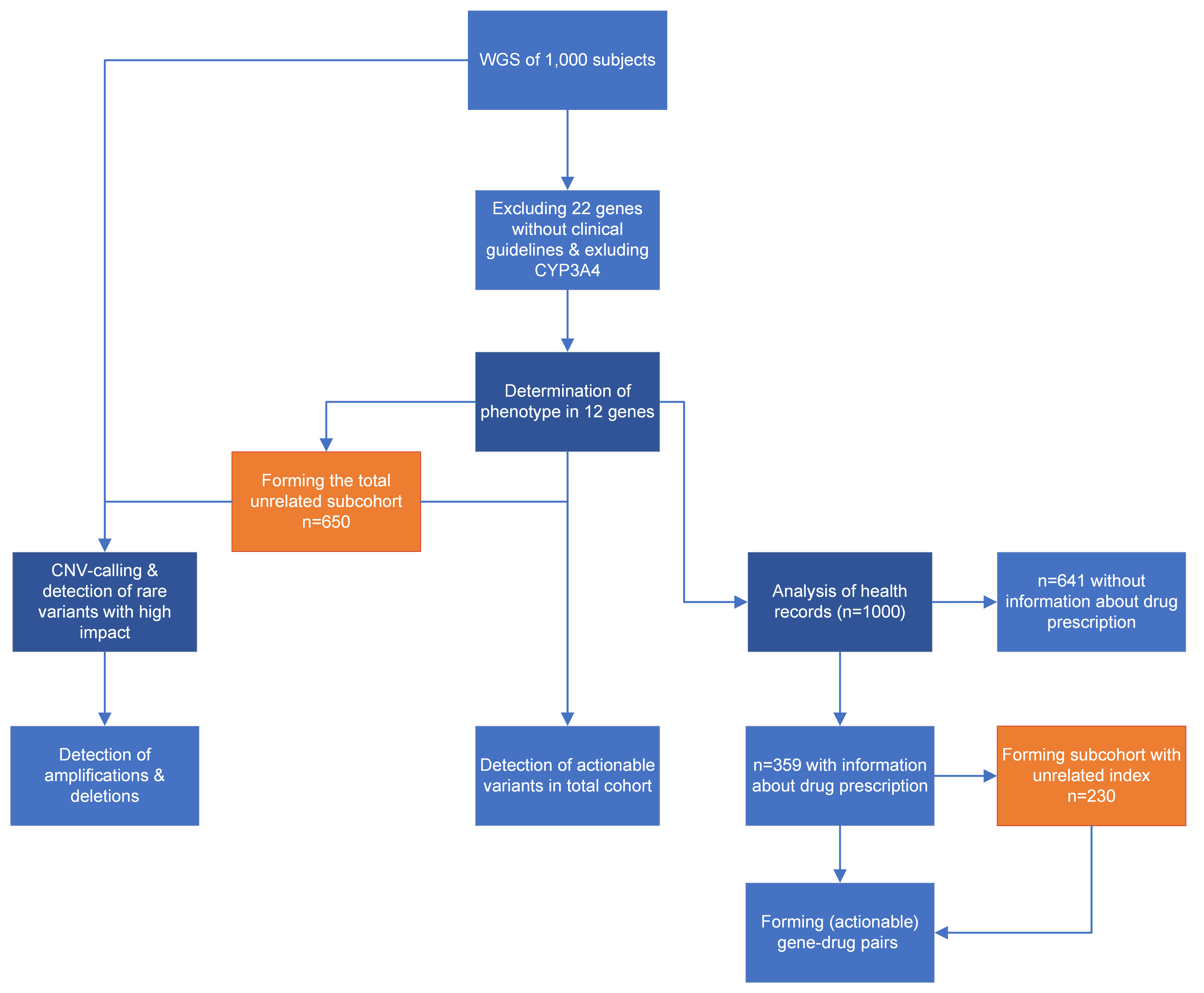

Supplement: Supplementary file 1 — Supplementary_figure_S1 [file 41431_2026_2025_MOESM1_ESM.png]

**A**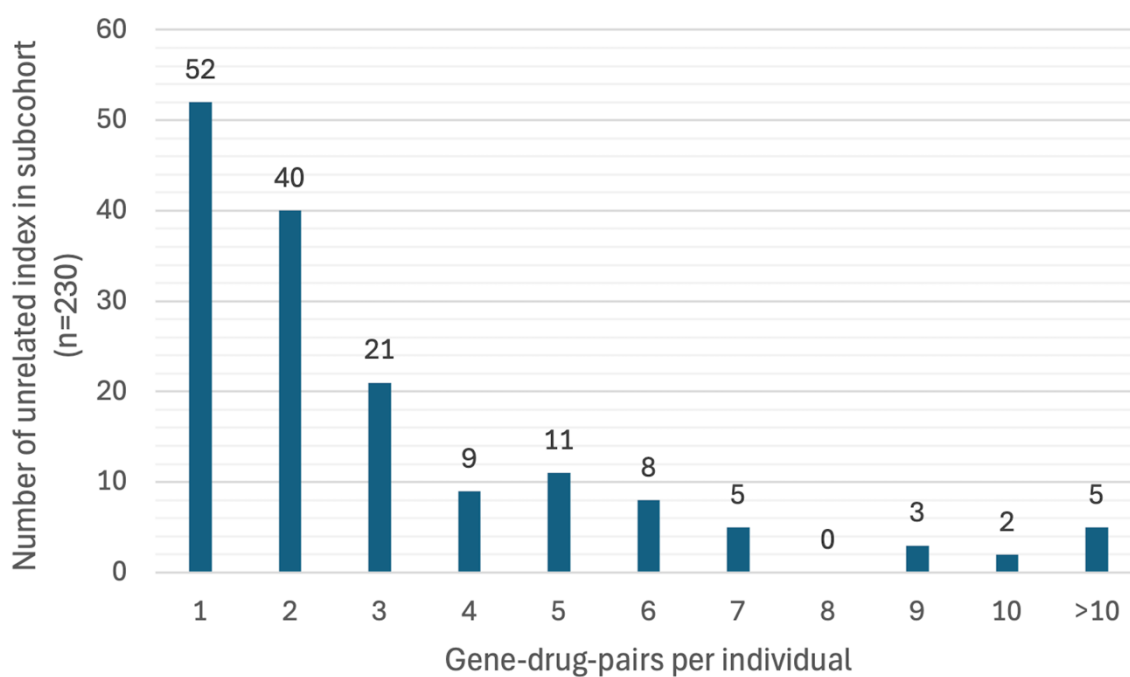**B**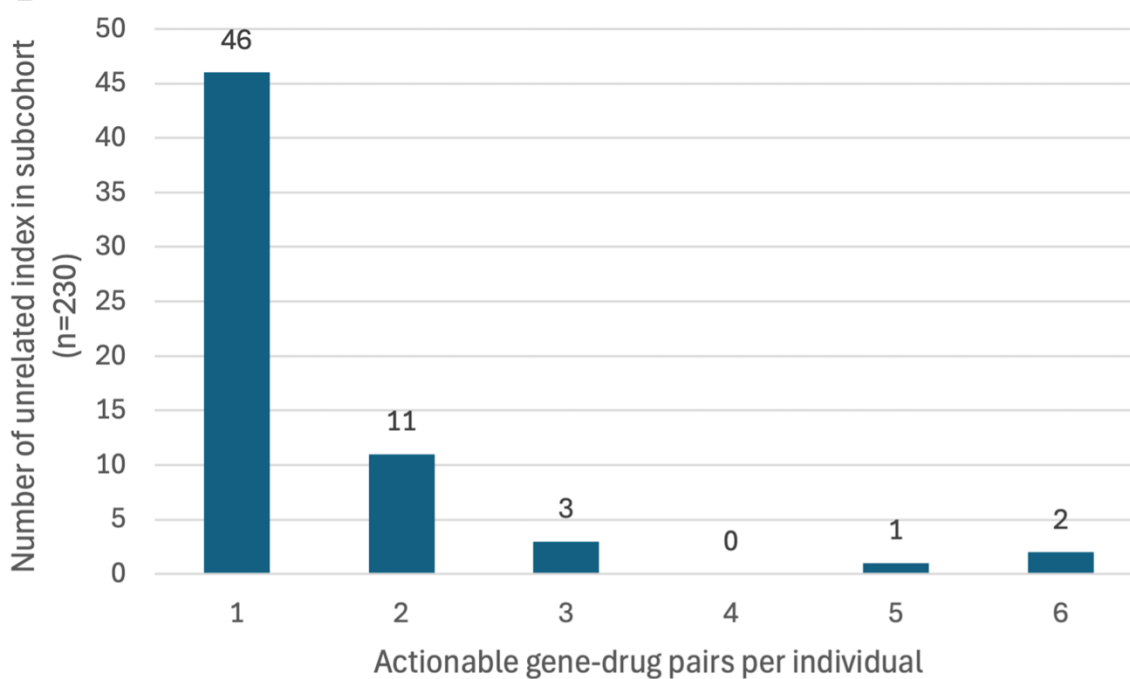

Supplement: Supplementary file 2 — Supplementary_Figure_S2 [file 41431_2026_2025_MOESM2_ESM.pdf]

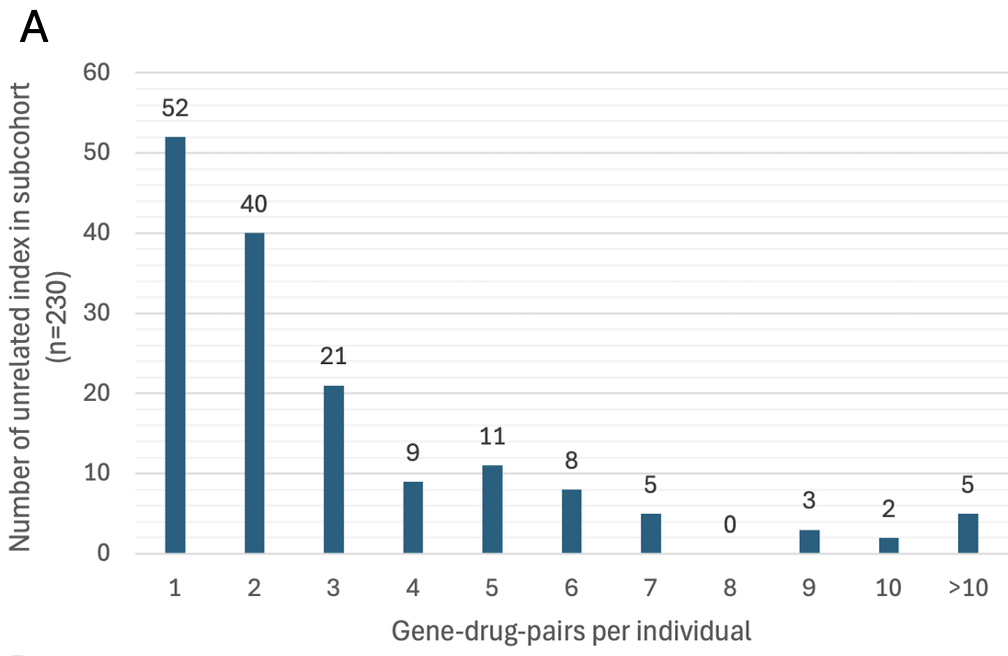

Supplement: Supplementary file 3 — Supplementary_figure_S2A [file 41431_2026_2025_MOESM3_ESM.png]

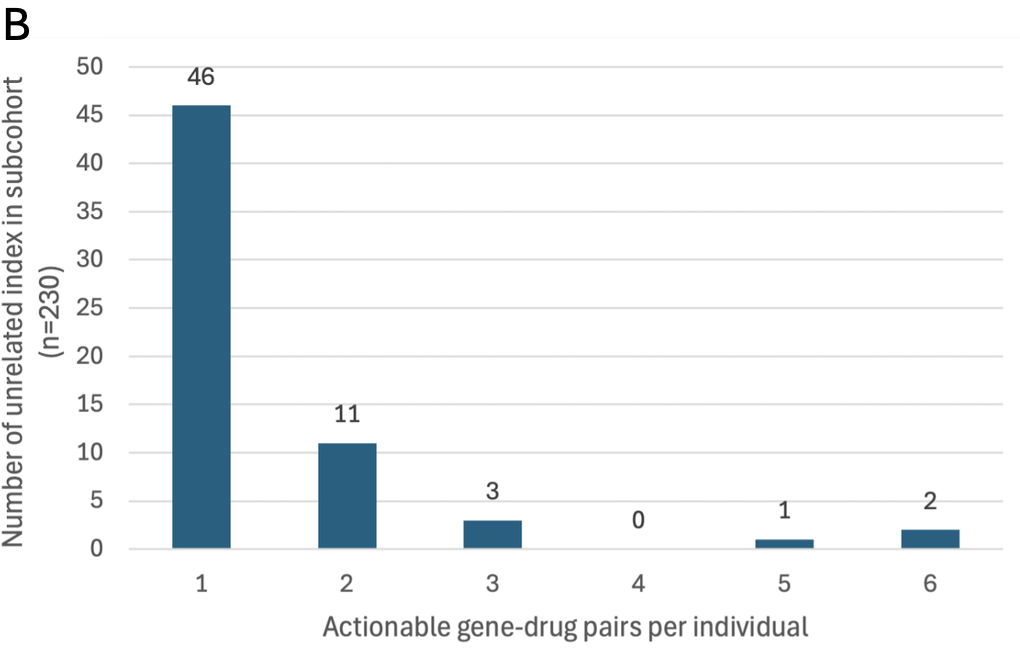

Supplement: Supplementary file 4 — Supplementary_figure_S2B [file 41431_2026_2025_MOESM4_ESM.png]

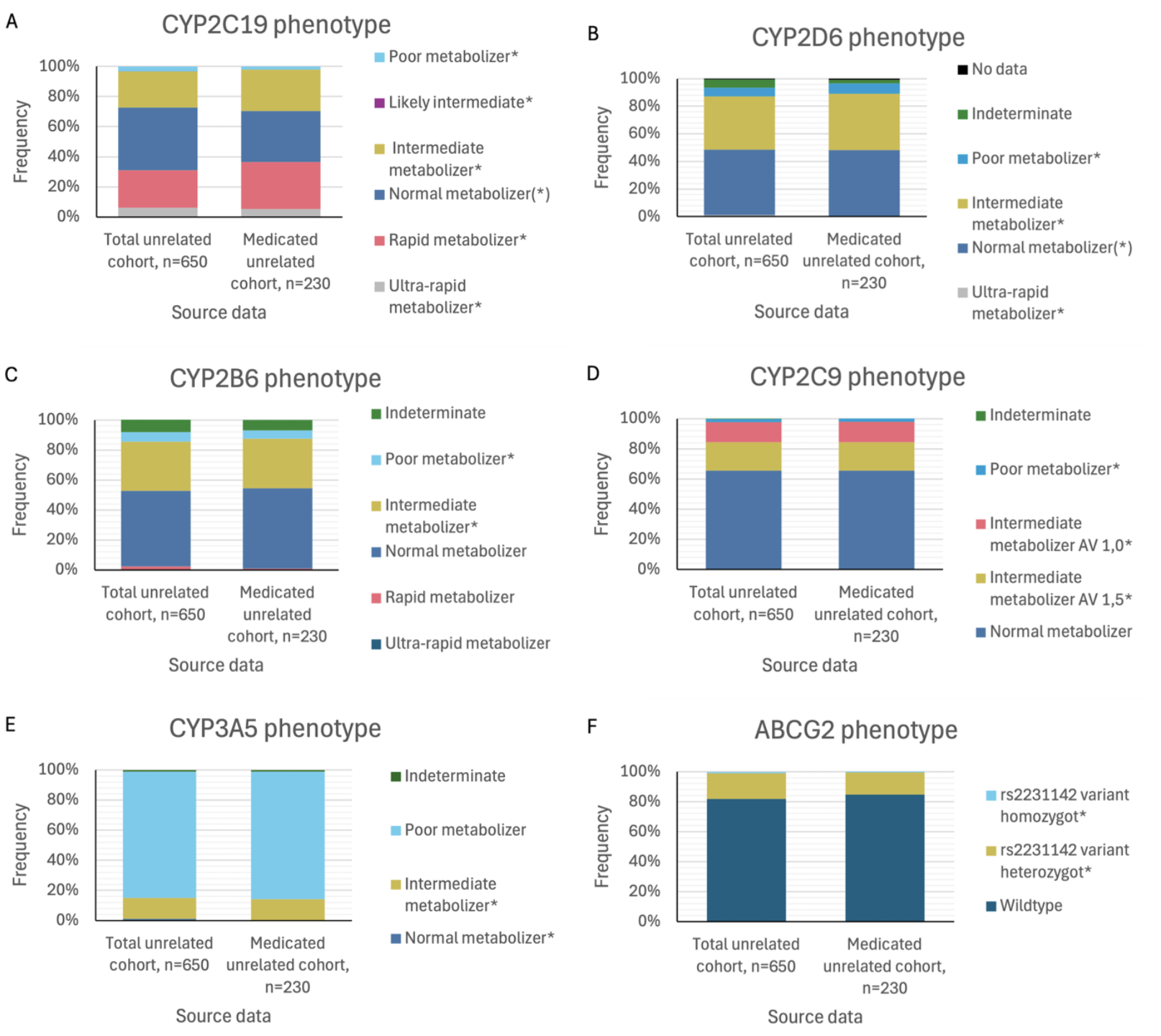

Supplement: Supplementary file 6 — Supplementary_figure_S3A [file 41431_2026_2025_MOESM6_ESM.png]

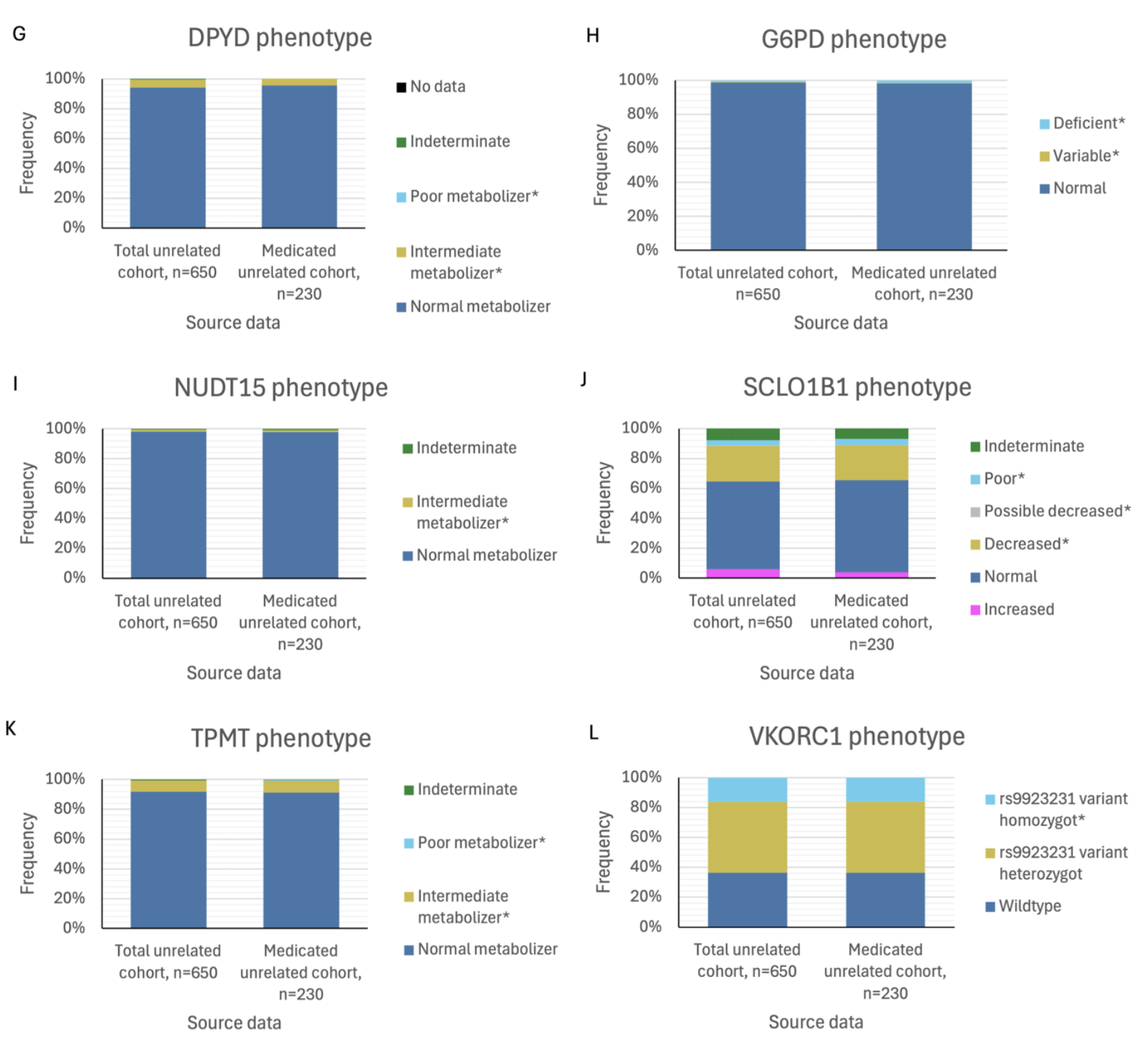

Supplement: Supplementary file 7 — Supplementary_figure_S3B [file 41431_2026_2025_MOESM7_ESM.png]
