# Supplementary material for: The potential of whole genome sequencing in pharmacogenetics: a retrospective health record study in rare disease patients
Source: Eur J Hum Genet. 2026 Feb 4;34(5):691–703. doi: 10.1038/s41431-026-02025-w (PMC13171899; doi:10.1038/s41431-026-02025-w)
Supplement: Supplementary file 5 — Supplementary_figure_S3 [file 41431_2026_2025_MOESM5_ESM.pdf]

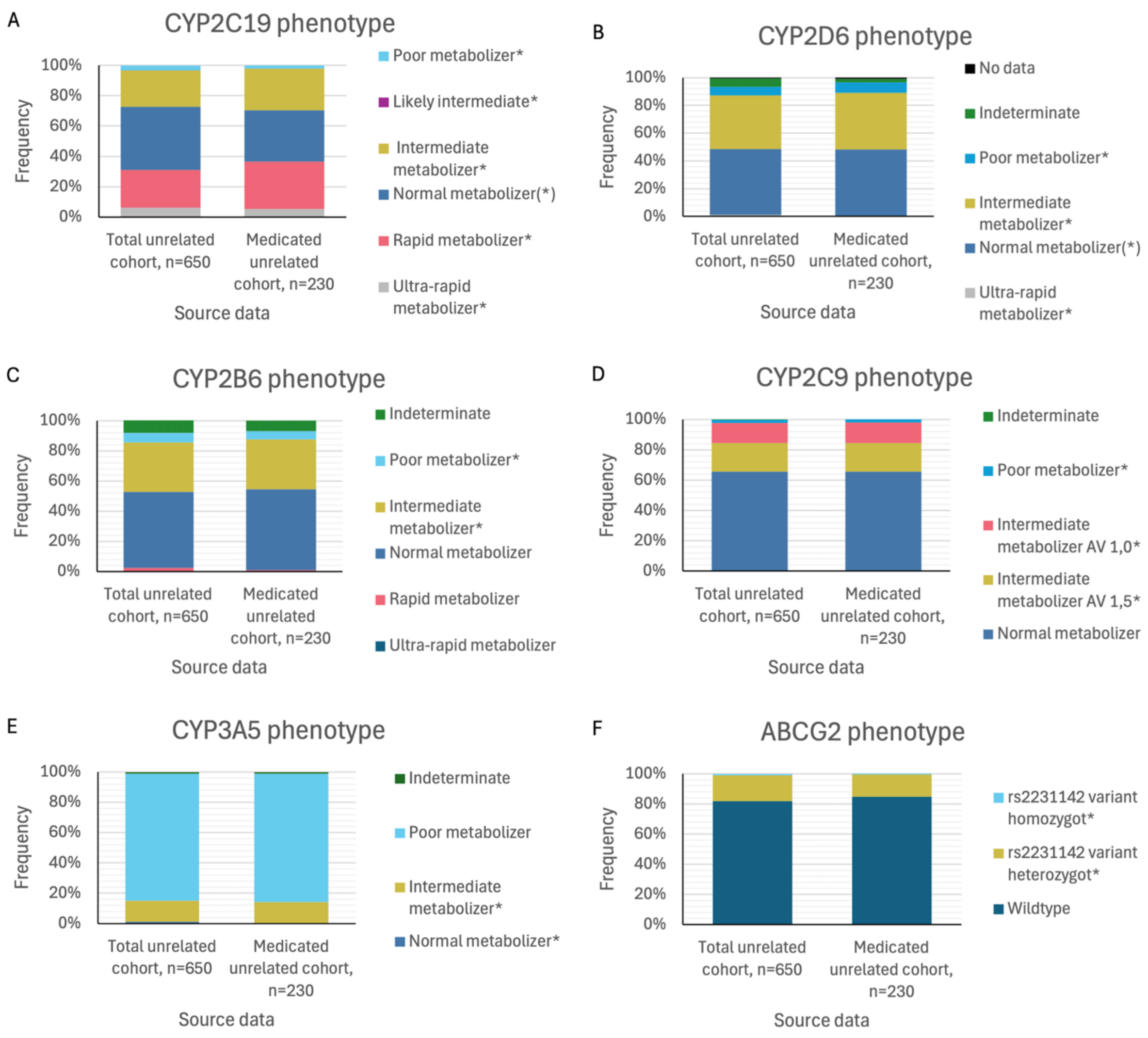
A
CYP2C19 phenotype
Frequency
100%
80%
60%
40%
20%
0%
Total unrelated cohort, n=650
Medicated unrelated cohort, n=230
Source data
Poor metabolizer*
Likely intermediate*
Intermediate metabolizer*
Normal metabolizer(*)
Rapid metabolizer*
Ultra-rapid metabolizer*
B
CYP2D6 phenotype
Frequency
100%
80%
60%
40%
20%
0%
Total unrelated cohort, n=650
Medicated unrelated cohort, n=230
Source data
No data
Indeterminate
Poor metabolizer*
Intermediate metabolizer*
Normal metabolizer(*)
Ultra-rapid metabolizer*
C
CYP2B6 phenotype
Frequency
100%
80%
60%
40%
20%
0%
Total unrelated cohort, n=650
Medicated unrelated cohort, n=230
Source data
Indeterminate
Poor metabolizer*
Intermediate metabolizer*
Normal metabolizer
Rapid metabolizer
Ultra-rapid metabolizer
D
CYP2C9 phenotype
Frequency
100%
80%
60%
40%
20%
0%
Total unrelated cohort, n=650
Medicated unrelated cohort, n=230
Source data
Indeterminate
Poor metabolizer*
Intermediate metabolizer AV 1,0*
Intermediate metabolizer AV 1,5*
Normal metabolizer
E
CYP3A5 phenotype
Frequency
100%
80%
60%
40%
20%
0%
Total unrelated cohort, n=650
Medicated unrelated cohort, n=230
Source data
Indeterminate
Poor metabolizer
Intermediate metabolizer*
Normal metabolizer*
F
ABCG2 phenotype
Frequency
100%
80%
60%
40%
20%
0%
Total unrelated cohort, n=650
Medicated unrelated cohort, n=230
Source data
rs2231142 variant homozygot*
rs2231142 variant heterozygot*
Wildtype

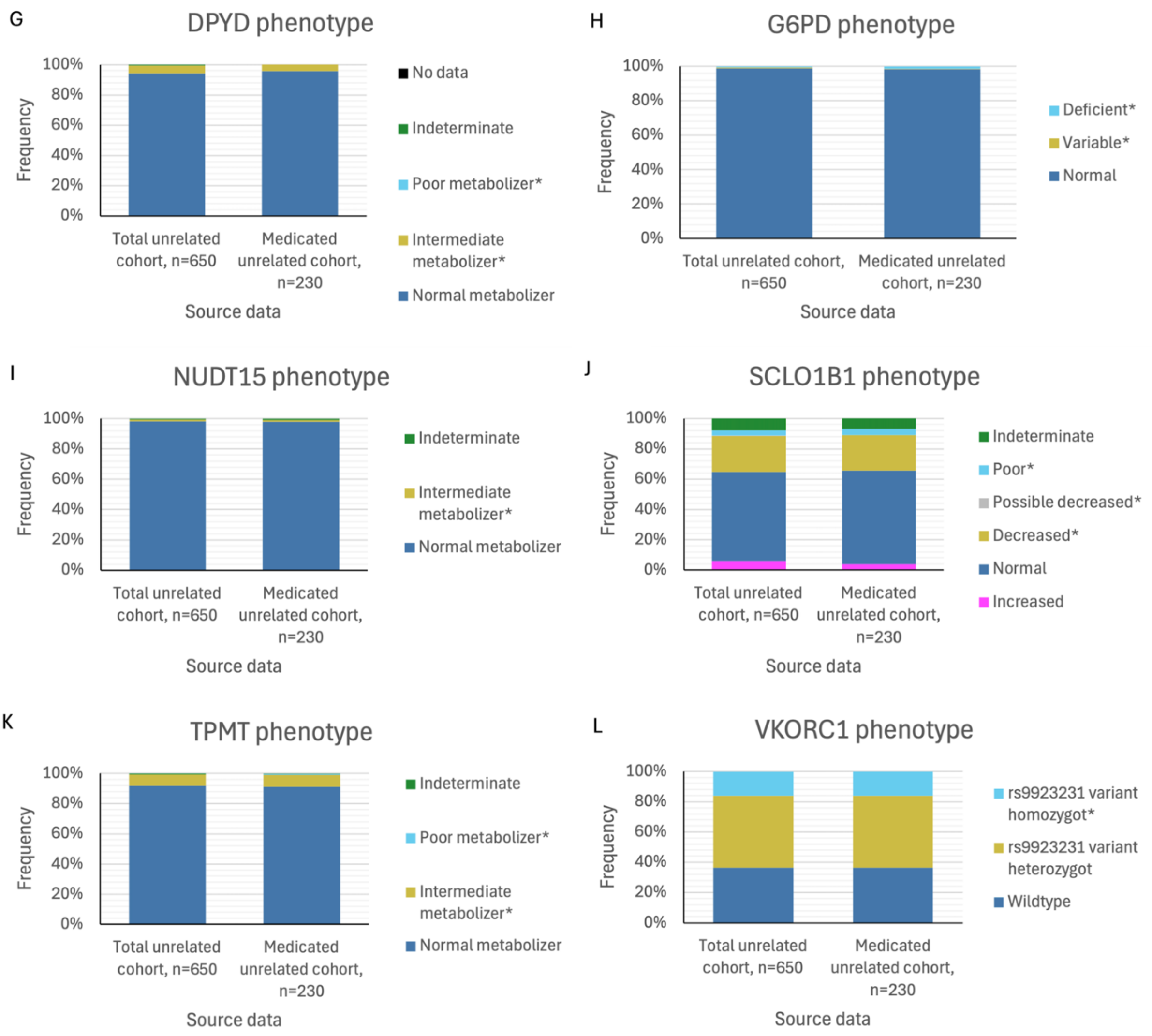
G
DPYD phenotype
Frequency
100%
80%
60%
40%
20%
0%
Total unrelated cohort, n=650
Medicated unrelated cohort, n=230
Source data
No data
Indeterminate
Poor metabolizer*
Intermediate metabolizer*
Normal metabolizer
H
G6PD phenotype
Frequency
100%
80%
60%
40%
20%
0%
Total unrelated cohort, n=650
Medicated unrelated cohort, n=230
Source data
Deficient*
Variable*
Normal
I
NUDT15 phenotype
Frequency
100%
80%
60%
40%
20%
0%
Total unrelated cohort, n=650
Medicated unrelated cohort, n=230
Source data
Indeterminate
Intermediate metabolizer*
Normal metabolizer
J
SCLO1B1 phenotype
Frequency
100%
80%
60%
40%
20%
0%
Total unrelated cohort, n=650
Medicated unrelated cohort, n=230
Source data
Indeterminate
Poor*
Possible decreased*
Decreased*
Normal
Increased
K
TPMT phenotype
Frequency
100%
80%
60%
40%
20%
0%
Total unrelated cohort, n=650
Medicated unrelated cohort, n=230
Source data
Indeterminate
Poor metabolizer*
Intermediate metabolizer*
Normal metabolizer
L
VKORC1 phenotype
Frequency
100%
80%
60%
40%
20%
0%
Total unrelated cohort, n=650
Medicated unrelated cohort, n=230
Source data
rs9923231 variant homozygot*
rs9923231 variant heterozygot
Wildtype
