# Supplementary material for: The potential of whole genome sequencing in pharmacogenetics: a retrospective health record study in rare disease patients
Source: Eur J Hum Genet. 2026 Feb 4;34(5):691–703. doi: 10.1038/s41431-026-02025-w (PMC13171899; doi:10.1038/s41431-026-02025-w)
Supplement: Supplementary file 8 — Supplementary_Table_S1_PGx [file 41431_2026_2025_MOESM8_ESM.pdf]

Table S1: Descriptive characteristics of the study population

	WGS cohort (n = 1,000)	Subcohort with WGS and prescription data (n = 359)
Age (in years), mean (SD)	36.5 (20.2)	32.5 (20.8)
Male sex, n(%)	499 (49.9)	172 (47.9)
Female sex, n(%)	501 (50.1)	187 (52.1)
Case solved(positive), n(%)	274 (27.4)	83 (23.1)
Index patient, n(%)	389 (38.9)	230 (64.1)
Family members of index patients, n(%)	612 (61.2)	129 (35.9)
Number of patients with rare disease symptoms, n(%)	448 (44.8)	248 (69.1)
Number of different families	389	259
**Indication for whole genome diagnostic, n(%)**		
Intellectual disability, n(%)	351 (35.1)	147 (40.1)
Neuromuscular disease, n(%)	193 (19.3)	79 (22.0)
Suspected Silver-Russel/Beckwith Wiedemann without molecular confirmation, n(%)	150 (15)	11 (3.1)
Tumor, n(%)	74 (7.4)	38 (10.6)
Epilepsy, n(%)	64 (6.4)	29 (8.1)
Retinopathy, n(%)	35 (3.5)	10 (2.8)
Skeletal dysplasia, n(%)	25 (2.5)	4 (1.1)
Polycystic kidney, n(%)	16 (1.6)	8 (2.2)
Ehlers-Danlos-/Marfan-syndrome, n(%)	16 (1.6)	7 (1.9)
Common Variable Immunodeficiency n(%)	16 (1.6)	6 (1.6)
Haematological Disorders, n(%)	14 (1.4)	6 (1.6)
Leukoencephalopathy, n(%)	12 (1.2)	3 (0.8)
Kardiomyopathy, n(%)	9 (0.9)	2 (0.6)
Hearing-Loss, n(%)	8 (0.8)	2 (0.6)
Mikrocephaly, n(%)	7 (0.7)	2 (0.6)
Rasopathy, n(%)	4 (0.4)	0 (0)
Cholestasis, n(%)	3 (0.3)	2 (0.6)
Syndromic disease, n(%)	3 (0.3)	1 (0.3)
No group, n(%)	3 (0.3)	2 (1.6)
