# Supplementary material for: The potential of whole genome sequencing in pharmacogenetics: a retrospective health record study in rare disease patients
Source: Eur J Hum Genet. 2026 Feb 4;34(5):691–703. doi: 10.1038/s41431-026-02025-w (PMC13171899; doi:10.1038/s41431-026-02025-w)
Supplement: Supplementary file 9 — Supplementary_Table_S2_PGx [file 41431_2026_2025_MOESM9_ESM.pdf]

Table S2 Distribution of phenotypes and frequencies by genes

	Subjects with whole genome sequencing, n = 1,000(%) (CPIC)	Subgroup of subjects with whole genome sequencing with available information on drug prescriptions, n = 359(%) (CPIC)
**CYP2C19 phenotype, n (%)**		
Ultra-rapid metabolizer*	52(5.2)	20(5.6)
Rapid metabolizer*	259(25.9)	106(29.5)
Normal metabolizer(*)	411(41.1)	123(34.3)
Intermediate metabolizer*	251(25.1)	103(28.7)
Likely intermediate*	1(0.1)	0(0.0)
Poor metabolizer*	26(2.6)	7(1.9)
Total with recommendation for possible treatment adjustment(%)	589(58.9)	236(65.7)
**CYP2D6 phenotype, n (%)**		
Ultra-rapid metabolizer*	15(1.5)	7(2)
Normal metabolizer(*)	477(47.7)	167(46.5)
Intermediate metabolizer*	378(37.8)	149(41.5)
Poor metabolizer*	65(6.5)	24(6.7)
Indeterminate	60(6.0)	9(2.5)
No data	5(0.5)	3(0.8)
Total with recommendation for possible treatment adjustment(%)	458(45.8)	180(50.1)
**CYP2B6 phenotype, n (%)**		
Ultra-rapid metabolizer	2(0.2)	0(0)
Rapid metabolizer	17(1.7)	3(0.8)
Normal metabolizer	498(49.8)	189(52.7)
Intermediate metabolizer*	335(33.5)	116(32.3)
Poor metabolizer*	66(6.6)	25(7.0)
Indeterminate	82(8.2)	26(7.2)
Total with recommendation for possible treatment adjustment(%)	401(40.1)	141(39.3)
**CYP2C9 phenotype, n (%)**		
Normal metabolizer	656(65.6)	239(66.6)

Intermediate metabolizer (AV 1.5)*	189	69
Intermediate metabolizer (AV1.0)*	132	42
Poor metabolizer*	22(2.2)	9(2.5)
Indeterminate	1(0.1)	0(0.0)
Total with recommendation for possible treatment adjustment(%)	343(34.3)	120(33.4)
**CYP3A4 phenotype, n (%)**		
Normal metabolizer	679(67.9)	238(66.3)
Intermediate metabolizer	58(5.8)	23(6.4)
Poor metabolizer*	1(0.1)	1(0.3)
No recommendation	262(26.2)	97(27.0)
Total with recommendation for possible treatment adjustment(%)	1(0.1)	1(0.3)
**CYP3A5 phenotype, n (%)**		
Normal metabolizer*	8(0.8)	1(0.3)
Intermediate metabolizer*	141(14.1)	50(13.9)
Poor metabolizer	841(84.1)	306(85.2)
No recommendation	10(1.0)	2(0.6)
Total with recommendation for possible treatment adjustment(%)	149(14.9)	51(14.2)
**ABCG2 phenotype, n (%)**		
Wildtype	815(81.5)	305(84.5)
rs2231142 variant heterozygous*	175(17.5)	52(14.5)
rs2231142 variant homozygote*	10(1.0)	2(1.0)
Total with recommendation for possible treatment adjustment(%)	185(18.5)	54(15.0)
**DPYD phenotype, n (%)**		
Normal metabolizer	945(94.5)	348(96.9)
Intermediate metabolizer*	51(5.1)	11(3.1)
Poor metabolizer*	0(0.0)	0(0.0)
Indeterminate	3(0.3)	0(0.0)
No data	1(0.1)	0(0.0)

Total with recommendation for possible treatment adjustment(%)	51(5.1)	11(3.1)
**G6PD phenotype, n (%)**		
Normal	986(98.6)	352(98.1)
Variable*	6(0.6)	3(0.8)
Deficient*	8(0.8)	4(1.1)
Total with recommendation for possible treatment adjustment(%)	14(1.4)	7(1.9)
**NUDT15 phenotype, n (%)**		
Normal metabolizer	982(98.2)	353(98.3)
Intermediate metabolizer*	11(1.1)	4(1.1)
Indeterminate	7(0.7)	2(0.6)
Total with recommendation for possible treatment adjustment(%)	11(1.1)	4(1.1)
**SCLO1B1 phenotype, n (%)**		
Increased	50(5.0)	20(5.6)
Normal	590(59.0)	218(60.7)
Decreased*	255(25.5)	87(24.2)
Possible decreased*	2(0.2)	1(0.3)
Poor*	31(3.1)	10(2.8)
Indeterminate	72(7.2)	23(6.4)
Total with recommendation for possible treatment adjustment(%)	288(28.8)	98(27.3)
**TPMT phenotype, n (%)**		
Normal metabolizer	913(91.3)	329(91.6)
Intermediate metabolizer*	77(7.7)	28(7.8)
Poor metabolizer*	2(0.2)	1(0.3)
Indeterminate	8(0.8)	1(0.3)
Total with recommendation for possible treatment adjustment(%)	79(7.9)	29(8.1)
**VKORC1 phenotype, n (%)**		
Wildtype	368(36.8)	141(39.3)
rs9923231 variant heterozygous	469(46.9)	158(44.0)
rs9923231 variant homozygote*	163(16.3)	60(16.7)

Total with recommendation for possible treatment adjustment(%)	163(16.3)	60(16.7)

*Phenotype, that could require treatment adjustment.

(*) If pantoprazole (CYP2C19) or atomoxetine (CYP2D6) included, normal metabolizer would require treatment adjustment.
