# Supplementary material for: The potential of whole genome sequencing in pharmacogenetics: a retrospective health record study in rare disease patients
Source: Eur J Hum Genet. 2026 Feb 4;34(5):691–703. doi: 10.1038/s41431-026-02025-w (PMC13171899; doi:10.1038/s41431-026-02025-w)
Supplement: Supplementary file 10 — Supplementary_Table_S3_PGx [file 41431_2026_2025_MOESM10_ESM.pdf]

Table S3: Incidence of relevant gene-gene-drug pairs detected by whole genome sequencing in the subgroup (n = 359)

Drug	Frequency drug-gene pair, n	Phenotype	Frequency per phenotype, n	Recommendation, based on ClinPGx	Frequencies of recommendation with action, n (%)
CYP2C19+CYP2D6					
Amitriptyline	10				6(60)
		NM+IM*	1	Dosing	
		IM+NM	4	No action	
		IM+IM*	1	Dosing	
		IM+UM*	1	Dosing/alternate drug	
		RM*+IM*	2	Dosing/alternate drug	
		RM*+NM	1	Dosing/alternate drug	
Doxepin	2	CYP2C19+CYP2D6			2(100)
		UM*+NM	1	Dosing/alternate drug	
		IM+IM*	1	Dosing	
Trimipramine	4	CYP2C19+CYP2D6			3(75)
		RM*+IM*	2	Dosing/alternate drug	
		IM+NM	1	No action	
		IM+IM*	1	Dosing	
CYP2C19+CYP2B6					
Sertraline	12	CYP2C19+CYP2B6			6(50)
		IM*+NM	1	Dosing	
		IM*+IM*	2	Dosing	
		IM*+PM*	1	Dosing	
		NM+IM*	2	Dosing	
		NM+NM	5	No action	
		RM+PM*	1	No action	
		RM+indeterminate	1	No action	
ABCG2+SCLO1B1					
Rosuvastatin	3	ABCG2+SCLO1B1			2(66.7)
		Normal+Poor function*	2	Dosing	
		Decreased+Normal function	1	No action	
NUDT15+TPMT		NUDT15+TPMT			
Azathioprine	3				0(0.0)
		NM+NM	3	No action	

Mercaptopurine	5				0(0.0)
		NM+NM	5	No action	
Thioguanine	3				0(0.0)
		NM+NM	3	No action	

: A phenotype marked with "" indicates that a therapy adjustment is recommended, when this phenotype is considered alone. If a phenotype is not marked with "*", no action is required and therapy with standard dose is recommended.
