# Supplementary material for: The potential of whole genome sequencing in pharmacogenetics: a retrospective health record study in rare disease patients
Source: Eur J Hum Genet. 2026 Feb 4;34(5):691–703. doi: 10.1038/s41431-026-02025-w (PMC13171899; doi:10.1038/s41431-026-02025-w)
Supplement: Supplementary file 11 — Supplementary_tableS4_PGx [file 41431_2026_2025_MOESM11_ESM.pdf]

Table S4 Distribution of phenotypes and frequencies by genes in unrelated sub cohorts

	Total unrelated, n = 650(%) (CPIC)	Unrelated index with medication information, n = 230(%) (CPIC)
**CYP2C19 phenotype, n (%)**		
Ultra-rapid metabolizer*	40(6.2)	12(5.2)
Rapid metabolizer*	161(24.8)	72(31.3)
Normal metabolizer(*)	272(41.8)	78(33.9)
Intermediate metabolizer*	155(23.8)	63(27.4)
Likely intermediate*	1(0.2)	0(0)
Poor metabolizer*	21(3.2)	5(2.2)
Total with recommendation for possible treatment adjustment(%)	378(58.2)	152(66.1)
**CYP2D6 phenotype, n (%)**		
Ultra-rapid metabolizer*	8(1.2)	1(0.4)
Normal metabolizer(*)	309(47.5)	110(47.8)
Intermediate metabolizer*	251(38.6)	94(40.9)
Poor metabolizer*	40(6.2)	17(7.4)
Indeterminate	39(6)	6(2.6)
No data	3(0.5)	2(0.9)
Total with recommendation for possible treatment adjustment(%)	299(46)	112(48.7)
**CYP2B6 phenotype, n (%)**		
Ultra-rapid metabolizer	1(0.2)	0(0)
Rapid metabolizer	13(2)	2(0.9)
Normal metabolizer	328(50.5)	123(53.5)
Intermediate metabolizer*	213(32.8)	76(33)
Poor metabolizer*	42(6.5)	13(5.7)
Indeterminate	53(8.2)	16(7)
Total with recommendation for possible treatment adjustment(%)	255(39.3)	89(38.7)
**CYP2C9 phenotype, n (%)**		

Normal metabolizer	427(65.7)	151(65.7)
Intermediate metabolizer (AV 1.5)*	121(18.6)	43(18.7)
Intermediate metabolizer (AV1.0)*	88(13.5)	31(13.5)
Poor metabolizer*	13(2)	5(2.2)
Indeterminate	1(0.2)	0(0)
Total with recommendation for possible treatment adjustment(%)	222(34.1)	79(34.4)
**CYP3A4 phenotype, n (%)**		
Normal metabolizer	440(67.7)	154(67)
Intermediate metabolizer	43(6.6)	13(5.7)
Poor metabolizer*	0(0)	1(0.4)
No recommendation	167(25.7)	62(27)
Total with recommendation for possible treatment adjustment(%)	0(0)	1(0.4)
**CYP3A5 phenotype, n (%)**		
Normal metabolizer*	7(1.1)	1(0.4)
Intermediate metabolizer*	90(13.8)	32(13.9)
Poor metabolizer	547(84.2)	195(84.8)
No recommendation	6(0.9)	2(0.9)
Total with recommendation for possible treatment adjustment(%)	97(14.9)	51(14.2)
**ABCG2 phenotype, n (%)**		
Wildtype	532(81.8)	195(84.8)
rs2231142 variant heterozygous*	112(17.2)	34(14.8)
rs2231142 variant homozygote*	6(0.9)	1(0.4)
Total with recommendation for possible treatment adjustment(%)	118(18.1)	35(15.2)
**DPYD phenotype, n (%)**		
Normal metabolizer	612(94.2)	220(95.7)
Intermediate metabolizer*	34(5.2)	10(4.3)
Poor metabolizer*	0(0)	0(0.0)
Indeterminate	3(0.5)	0(0.0)

No data	1(0.2)	0(0.0)
Total with recommendation for possible treatment adjustment(%)	34(5.2)	10(4.3)
**G6PD phenotype, n (%)**		
Normal	642(98.8)	226(98.3)
Variable*	4(0.6)	1(0.4)
Deficient*	4(0.6)	3(1.3)
Total with recommendation for possible treatment adjustment(%)	8(1.2)	4(1.7)
**NUDT15 phenotype, n (%)**		
Normal metabolizer	639(98.3)	225(97.8)
Intermediate metabolizer*	6(0.9)	3(1.3)
Indeterminate	5(0.8)	2(0.9)
Total with recommendation for possible treatment adjustment(%)	6(0.9)	3(1.3)
**SCLO1B1 phenotype, n (%)**		
Increased	38(5.8)	9(3.9)
Normal	383(58.9)	142(61.7)
Decreased*	154(23.7)	54(23.5)
Possible decreased*	2(0.3)	0(0)
Poor*	23(3.5)	9(3.9)
Indeterminate	50(7.7)	16(7)
Total with recommendation for possible treatment adjustment(%)	179(27.5)	63(27.4)
**TPMT phenotype, n (%)**		
Normal metabolizer	596(91.7)	210(91.3)
Intermediate metabolizer*	49(7.5)	18(7.8)
Poor metabolizer*	0(0)	1(0.4)
Indeterminate	5(0.8)	1(0.4)
Total with recommendation for possible treatment adjustment(%)	49(7.5)	19(8.2)
**VKORC1 phenotype, n (%)**		
Wildtype	238(36.6)	84(36.5)
rs9923231 variant heterozygous	310(47.7)	109(47.4)

rs9923231 variant homozygote*	102(15.7)	37(16.1)
Total with recommendation for possible treatment adjustment(%)	102(15.7)	37(16.1)

*Phenotype, that could require treatment adjustment.

(*) If pantoprazole (CYP2C19) or atomoxetine (CYP2D6) included, normal metabolizer would require treatment adjustment.
